# The volatile anesthetic isoflurane differentially inhibits voltage-gated sodium channel currents between pyramidal and parvalbumin neurons in the prefrontal cortex

**DOI:** 10.3389/fncir.2023.1185095

**Published:** 2023-06-16

**Authors:** Jingxuan Qiu, Yaoxin Yang, Jin Liu, Wenling Zhao, Qian Li, Tao Zhu, Peng Liang, Cheng Zhou

**Affiliations:** ^1^Department of Anesthesiology, West China Hospital of Sichuan University, Chengdu, China; ^2^Laboratory of Anesthesia and Critical Care Medicine, National-Local Joint Engineering Research Centre of Translational Medicine of Anesthesiology, West China Hospital of Sichuan University, Chengdu, China; ^3^Department of Anesthesiology, Zhongshan Hospital, Fudan University, Shanghai, China

**Keywords:** voltage-gated sodium channel (Na_v_), isoflurane, pyramidal neurons, parvalbumin neurons, cortex

## Abstract

**Background:**

How volatile anesthetics work remains poorly understood. Modulations of synaptic neurotransmission are the direct cellular mechanisms of volatile anesthetics in the central nervous system. Volatile anesthetics such as isoflurane may reduce neuronal interaction by differentially inhibiting neurotransmission between GABAergic and glutamatergic synapses. Presynaptic voltage-dependent sodium channels (Na_v_), which are strictly coupled with synaptic vesicle exocytosis, are inhibited by volatile anesthetics and may contribute to the selectivity of isoflurane between GABAergic and glutamatergic synapses. However, it is still unknown how isoflurane at clinical concentrations differentially modulates Na_v_ currents between excitatory and inhibitory neurons at the tissue level.

**Methods:**

In this study, an electrophysiological recording was applied in cortex slices to investigate the effects of isoflurane on Na_v_ between parvalbumin (PV^+^) and pyramidal neurons in PV-cre-tdTomato and/or vglut2-cre-tdTomato mice.

**Results:**

Isoflurane at clinically relevant concentrations produced a hyperpolarizing shift in the voltage-dependent inactivation and slowed the recovery time from the fast inactivation in both cellular subtypes. Since the voltage of half-maximal inactivation was significantly depolarized in PV^+^ neurons compared to that of pyramidal neurons, isoflurane inhibited the peak Na_v_ currents in pyramidal neurons more potently than those of PV^+^ neurons (35.95 ± 13.32% vs. 19.24 ± 16.04%, *P* = 0.036 by the Mann-Whitney test).

**Conclusions:**

Isoflurane differentially inhibits Na_v_ currents between pyramidal and PV^+^ neurons in the prefrontal cortex, which may contribute to the preferential suppression of glutamate release over GABA release, resulting in the net depression of excitatory-inhibitory circuits in the prefrontal cortex.

## Introduction

The clinical application of volatile anesthetics has been around for more than 170 years, and they induce many clinically necessary pharmacological actions, including amnesia, unconsciousness, and immobility (Hemmings et al., 2005). However, the exact cellular and/or molecular mechanism of how volatile anesthetics work remains poorly understood (Hemmings et al., 2005). Synaptic neurotransmission is the basic neural function for passing information to the central nervous system (Hao et al., 2020), and volatile anesthetics have been known to modulate synaptic neurotransmission at both presynaptic and postsynaptic levels (Hemmings et al., 2005; Westphalen and Hemmings, 2006). Volatile anesthetics may reduce neuronal interaction by inhibiting neurotransmission. Therefore, investigating the specific synaptic targets for volatile anesthetics is critical for understanding anesthetic mechanisms and developing novel and selective general anesthetics. There is a relatively good understanding regarding the effects of volatile anesthetics on postsynaptic neurotransmitter receptors, mainly including the facilitation of inhibitory GABA_A_ receptors and the inhibition of excitatory *N*-methyl-D-aspartate (NMDA) receptors (Hao et al., 2020). Compared to postsynaptic modulations, volatile anesthetics have been shown to suppress presynaptic neurotransmitter release (Schlame and Hemmings, 1995; Westphalen et al., 2013), and these effects are mainly mediated by their suppressions in presynaptic voltage-gated sodium channels (Na_v_) and voltage-gated calcium channels (Ca_v_) (Wu et al., 2004; Hao et al., 2020).

Previously, volatile anesthetics were found to suppress excitatory synaptic transmission (glutamate) more potently than GABA release on cultured hippocampal neurons in an action potential-dependent way, which indicates that the presynaptic Na_v_ may be involved (Speigel and Hemmings, 2021). By selectively targeting excitatory and inhibitory neurotransmitters, volatile anesthetics may produce a net depression effect within the pyramidal-interneuron microcircuits (Maclver et al., 1996; Westphalen et al., 2013). At the behavioral level, Na_v_ has emerged as an underlying target for the pharmacological actions of volatile anesthetics. For example, the intrathecal administration of the highly specific Na_v_ antagonist tetrodotoxin (TTX) in adult rats enhances the immobility potency of isoflurane, whereas co-administration of the Na_v_ agonist veratridine reduces isoflurane potency and counteracts the effect of TTX (Zhang et al., 2010). At the molecular level, it has been found that the volatile anesthetic inhibits Na_v_ currents both in transfected cells and hippocampal brain slices at their clinically relevant concentrations (Rehberg et al., 1996; Purtell et al., 2015; Zhou et al., 2019). Interestingly, recent studies have indicated that the volatile anesthetic isoflurane inhibits Na_v_1.6 more potently than Na_v_1.1 at resting membrane potentials because of the varied voltage-dependent inactivation between the Na_v_ subtypes in transfected cells (Zhou et al., 2019). It is worth noting that parvalbumin (PV^+^) neurons are enriched in Na_v_1.1 (Hu and Jonas, 2014; Li et al., 2014), whereas glutamatergic neurons are more abundant in Na_v_1.6 (Speigel and Hemmings, 2021). Accordingly, Speigel and Hemmings (2021) revealed that the differential expression of the Na_v_ subtype between glutamatergic and PV^+^ neurons contributed to the stronger inhibition of presynaptic glutamate by isoflurane compared to the GABA release in primary hippocampal neurons. However, the above results that isoflurane inhibits synaptic vesicle exocytosis and neurotransmitter release were mainly investigated in isolated nerve terminals or cultured primary neurons *in vitro*. It is still unknown whether isoflurane at clinical concentrations differentially modulates Na_v_ currents and action potentials between excitatory vs. inhibitory neurons at the tissue level, especially in brain regions associated with unconsciousness induced by general anesthetics.

An increasing number of studies have reported that the prefrontal cortex (PFC), especially layer 5, may be the key neural substrate relevant to unconsciousness induced by general anesthetics (Briner et al., 2010; Guidera et al., 2017; Suzuki and Larkum, 2020). Pyramidal neurons are the dominant excitatory neurons in the PFC, while parvalbumin (PV^+^) neurons are the critical GABAergic interneurons (Ährlund-Richter et al., 2019; Bhattacherjee et al., 2019). Therefore, in this study, we combined brain slice electrophysiological recordings and simulation *in silico* to test the hypothesis that volatile anesthetic isoflurane may inhibit Na_v_ currents more potently in excitatory pyramidal neurons than inhibitory PV^+^ neurons, which may contribute to the net depression within the pyramidal-interneuron microcircuits in the cortex.

## Materials and methods

### Animals

The experimental protocol was performed in strict adherence to the guidelines of Animal Research Reporting of *In Vivo* Experiments (ARRIVE) and approved by the Animal Ethics Committee of West China Hospital of Sichuan University (Chengdu, China) (No. 2021177A). PV-Cre knockin mice (strain number 017320, Jackson Laboratory) have Cre recombinase expressed in parvalbumin-expressing neurons, and glut2-ires-Cre knockin mice (strain number 028863, Jackson Laboratory) express Cre recombinase in excitatory glutamatergic neurons, where Cre expression is controlled by the endogenous vesicular glutamate transporter 2 (vglut2) gene promotor, a glutamatergic neuron marker. To identify prefrontal cortex PV^+^ (parvalbumin) neurons and/or glutamatergic neurons, we crossed PV-cre mice and/or glut2-Cre mice with Ai9 mice (strain number 007909, Jackson Laboratory), which expressed robust tdTomato fluorescence following Cre-mediated recombination. Adult vglut2-cre-tdTomato mice and/or PV-cre-tdTomato mice (>8 weeks old) were housed under standard conditions with a 12-h (7:00–19:00) light/dark cycle at constant humidity (45–55%) and temperature (22–24°C) with free access to food and water. The female and male mice were both used for all experiments and were randomly assigned to the experimental groups.

### Preparation of an acute brain slice

Adult mice were anesthetized with 2% isoflurane. Their brains were quickly dissected, and transverse slices (300 μm in thickness) containing the PFC region were obtained. The process was carried out in an ice-cold cutting solution containing the following components (in mM): 260 sucrose, 26 NaHCO_3_, 3 KCl, 1.25 NaH_2_PO_4_, 1 CaCl_2_, 5 MgCl_2_, and 10 glucose. A vibratome was used to obtain the slices (VT1000 A; Leica Microsystems Inc., Buffalo Grove, IL, USA). The slices were immediately transferred and incubated at 35–37°C with an external solution containing (in mM) 130 NaCl, 3 KCl, 2 MgCl_2_, 2 CaCl_2_, 1.25 NaH_2_PO_4_, 26 NaHCO_3_, and 10 glucose for 30 min and then maintained at room temperature (24–26°C) for 30 min before recording. The brain slices in the incubation solution were continuously bubbled with 95% O_2_/5% CO_2_ (pH = 7.35).

### Electrophysiological recording

Each brain slice containing the PFC regions was mounted in a recording chamber submerged in a continuously perfused external solution at a rate of ~2 ml/min bubbled with 95% O_2_/5% CO_2_, pH = 7.35. Electrophysiological recordings (24–26°C) were conducted at room temperature using an Axopatch 700B amplifier and a Digidata1440 digitizer linked to a computer running pClamp 10.6 software (Molecular Devices, Sunnyvale, CA, USA). The signals were recorded at 20 kHz and filtered at 10 kHz.

Whole-cell current-clamp and voltage-clamp recordings were established using pipettes with a resistance of 4–5 MΩ and made from the soma of PV^+^ and/or pyramidal neurons in layer 5 of the PFC region. Na_v_-mediated currents were measured in whole-cell voltage-clamp using cesium (Cs^+^)-based internal solutions (in mM): 104 Cs-CH_3_SO_3_, 1 MgCl_2_, 0.5 CaCl_2_, 30 tetraethylammoniums (TEA)-Cl, 10 EGTA, 3 Mg-ATP, 0.3 GTP-Tris, 10 HEPES (pH = 7.2, adjusted with CsOH). The external solution was added with TEA-Cl (20 mM), 4-aminopyridine (5 mM), bicuculline (10 μM), picrotoxin (100 μM), and cyanquixaline (10 μM) to block K_v_ and synaptic/extrasynaptic GABAergic and glutamatergic signaling. The current-voltage (I-V) relationship of Na_v_ currents was determined using voltage steps between −70 and +70 mV (10 mV step). Cell and electrode capacitances were electronically compensated during the recording. The initial access resistance was <15 MΩ, and the cell was discarded if the resistance changed by >25% during the recording. In our experimental protocol, we showed the Na_v_ currents before and after electronic compensation and then perfused them with 200 nM TTX to identify the properties of the channel recorded.

Action potential (AP) was elicited in response to depolarizing current steps from 0 to 300 pA (30 pA step, 1,000 ms) in the current-clamp mode using a K^+^-based internal solution (in mM): 130 KCl, 2 NaCl, 10 HEPES, 5 EGTA, 2 Mg-ATP, and 0.5 CaCl_2_ (pH = 7.2, adjusted using KOH). Resting membrane potential (RMP) was recorded as the voltage with no injected current (*I* = 0). Input resistance (R_in_) was calculated as the slope of the linear portion of the voltage-current curve responding to hyperpolarizing current injections from −120 up to −60 pA in 30 pA increments and 1,000 ms duration. The properties (amplitudes, widths at half-maximum, and dv/dt) of the action potential were analyzed using Clampfit 10.6 software.

### Preparation of volatile anesthetics

Isoflurane was obtained from Abbott Pharmaceutical Co., Ltd. (China). Saturated stock solutions of isoflurane (10–12 mM) were confirmed using gas chromatography and prepared by adding liquid isoflurane into an artificial cerebrospinal fluid and rotating up and down in gas-tight glass bottles for at least 24 h before use. The desired final concentrations of isoflurane were prepared by diluting the saturated stock solution with artificial cerebrospinal fluid. Finally, 0.30 mM of isoflurane (at 25°C) was used as the predicted minimum alveolar concentration (MAC) for mice (Franks and Lieb, 1996).

### Simulation of the effects of isoflurane on APs and synaptic currents *in silico*

A computational model using NEURON software 7.4 (http://www.neuron.yale.edu/neuron/) was used to simulate the effects of isoflurane on AP frequency and synaptic release on PV^+^ and pyramidal neurons. Simulation of presynaptic APs was modified from the computational model (Akemann et al., 2009) to be mediated by Na_v_ properties, with soma length and width set at 25 mm and Ra at 80 V/cm. The electrophysiological properties of the original pas (passive) and hh (Hodgkin-Huxley) channels were set to default NEURON values, and the resting membrane potential of the soma was set at −70 mV. The differential APs frequencies on PV^+^ and/or pyramidal neurons (the frequency of APs on PV^+^ neurons was faster than that on pyramidal neurons) were simulated by adjusting Na_v_ electrophysiological parameters (Herzog et al., 2001) according to the varied parameters we obtained in brain slice patch-clamping recordings. The control presynaptic stimulus was 100 ms at 100 Hz. The relationship between AP amplitude and the probability of transmitter release was modeled using a previously established nerve terminal model (Graham and Redman, 1994). As mentioned in the results section, voltage-dependent inactivation of Na_v_ was more depolarized in PV^+^ neurons, and its steady-state recovery was much faster. Therefore, the baseline frequency of APs was faster in PV^+^ neurons compared to pyramidal neurons by changing the Na_v_ parameters. The simulation of postsynaptic currents was modified from Graham et al. (2001); the post-synaptic currents were determined by both presynaptic AP frequency and amplitudes, and it was assumed they were both excitatory transmissions. The effects of isoflurane in this simulation were also based on our previous recordings in brain slices. The detailed adjustments to parameters are listed in the results section of the simulation.

### Statistical analysis

Measurement data were expressed as mean ± standard error (mean ± SEM). GraphPad Prism software (version 9.0; San Diego, CA, USA) was used for statistical analysis, and Clampfit 10.6 software was used for data extraction and analysis of patch-clamp results. The data were tested for normality using the Shapiro–Wilk test. When the data conformed to a normal distribution, the comparison between the two groups was performed using a paired or unpaired *t*-test; otherwise, the Mann–Whitney test or the Wilcoxon test was used. Repeated measures data were analyzed using one-way or two-way analysis of variance (ANOVA) with a Bonferroni *post-hoc* test. The exact statistical method used for each comparison is described in the figure legends. Statistical significance was set at a *P-*value of < 0.05.

## Results

### Voltage-dependent activations of Na_v_ are similar between the PV^+^ and pyramidal neurons in the PFC, while steady-state inactivation and recovery are different

**Figure 1:**
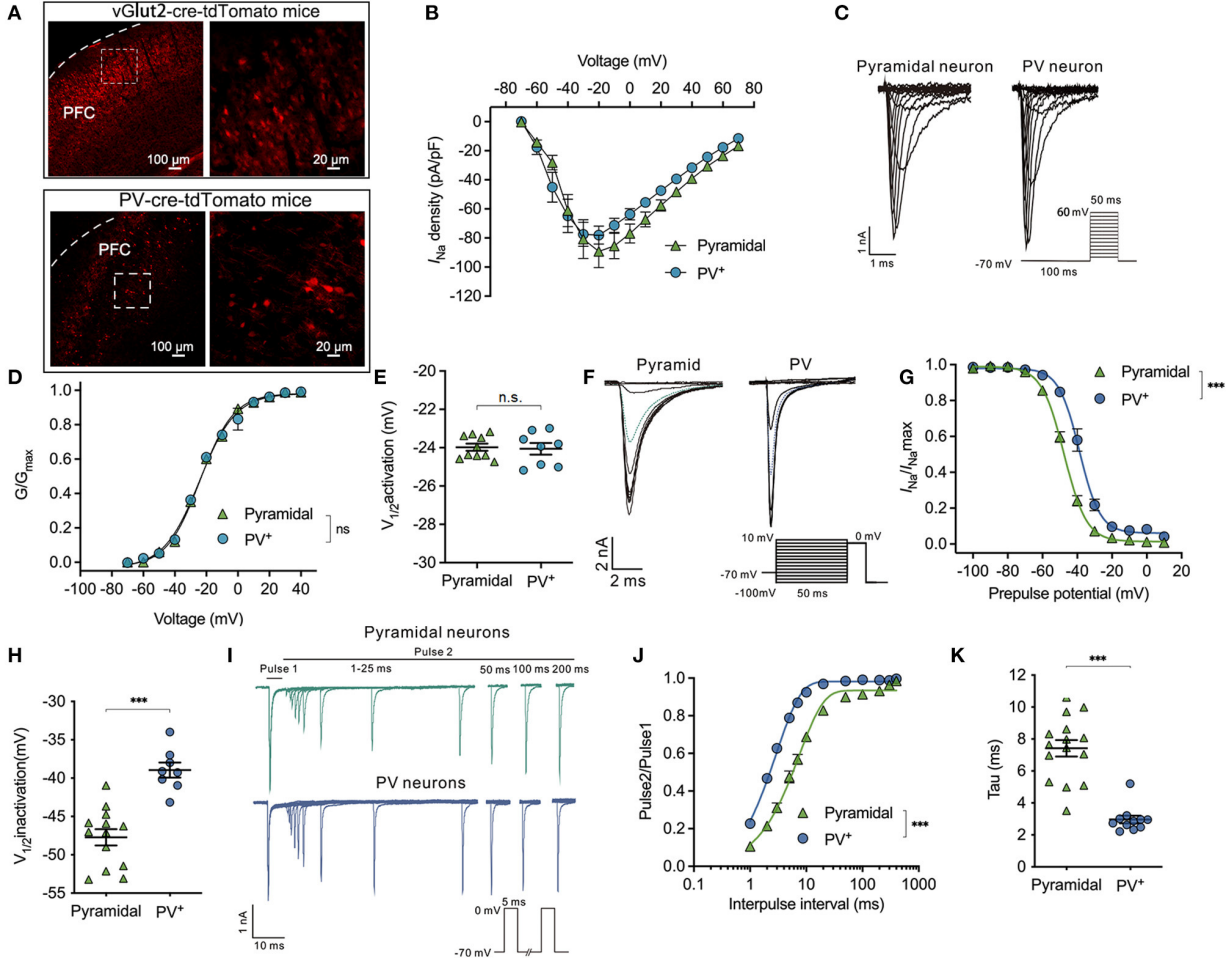
Difference in properties of Na_v_ activation, inactivation, and recovery between PV^+^ and pyramidal neurons. **(A)** Representative images of tdTomato fluorescent (red) to identify the parvalbumin (PV^+^) and pyramidal neurons in vglut2-cre-tdTomato (upper) and/or PV-cre-tdTomato (down) mice in the cortex. The right images are the enlargement of the left white frame enclosed part. **(B)** Current-voltage relationship recorded from −70 to +70 mV between the pyramidal and PV^+^ neurons. **(C)** A representative trace for the activation of Na_v_ in response to −70 to +60 mV between the pyramidal and PV^+^ neurons. **(D, E)** Activation curves for Na_v_ **(D)** and the voltage of half-maximum activation (V_1/2activation_) **(E)** are similar between the pyramidal and PV^+^ neurons (*n* = 8–10 cells from 4 to 5 mice). **(F)** A representative trace for the voltage-dependent inactivation of Na_v_ in response to −100 to +10 mV using the double pulse between the pyramidal and PV^+^ neurons. **(G, H)** Inactivation curves for Na_v_ **(G)** and voltage of half-maximum inactivation (V_1/2inactivation_) **(H)** recorded in PV^+^ neurons shift to a depolarized direction compared with pyramidal neurons (*n* = 15–17 cells from 6 to 7 mice). **(I)** Representative trace for the recovery of Na_v_ between pyramidal and PV^+^ neurons. **(J, K)** Normalized peak current (Pulse2/Pulse1) plotted against duration of the inter-pulse interval for Na_v_ **(J)** and recovery time constant (tau) from inactivation **(K)** (*n* = 15 cells from 7 to 8 mice). Data are presented as means ± SEM. ***P < 0.001; n.s., not significant.

To identify the PV^+^ and/or pyramidal neurons in the PFC, PV-Cre mice and/or vglut2-cre mice were crossed with Ai9 mice, which expressed tdTomato fluorescence following Cre-mediated recombination. In PV-cre-tdTomato mice and/or vgult2-cre-tdTomato mice, the glutamatergic neurons (Figure 1A, top) and/or PV^+^ neurons (Figure 1A, down) were labeled with robust tdTomato fluorescence. The current-voltage (I-V) relationship of Na_v_ was determined by a series of 50 ms voltage steps from −70 to +70 mV with a holding potential (V_holding_) of −70 mV. The peak *I*_Na_ was recorded at approximately −20 mV in both types of neurons (Figure 1B). The voltage-dependent activation of Na_v_ was determined using a 100-ms conditioning pulse to −100 mV and immediately following a series of 50 ms voltage steps from −70 to +60 mV (Figure 1C). Then, G/G_max_ was the normalized fractional conductance reflected by the fraction of Na_v_ activated during the test pulse. The voltage-dependent activation of Na_v_ was not different between PV^+^ neurons and pyramidal neurons (using a two-way ANOVA, *P* = 0.263 for interaction between groups × time, *F*_(11,176)_ = 1.241; *P* < 0.001 for time, *F*_(1.415, 22.64)_ = 1,996; *P* = 0.934 for PV^+^ vs. pyramidal neurons, *F*_(1,16)_ = 0.737; *n* = 8–10, Figure 1D). Therefore, the voltages at which currents of activation were half-maximal (V_1/2activation_) were compared between PV^+^ and pyramidal neurons, and the values of V_1/2activation_ were similar between the two types of neurons (−24.06 ± 0.87 vs. −23.98 ± 0.60 mV, *P* = 0.821 by unpaired *t*-test, Figure 1E). All the above results indicate that the activation properties of Na_v_ are similar between PV^+^ neurons and pyramidal neurons in the PFC region.

Next, the properties of inactivation and recovery of Na_v_ channels were recorded. The steady-state inactivation was determined using a double pulse protocol, which comprised a 300-ms conditioning pulse ranging from −100 to +10 mV (step = 10 mV), followed immediately by a depolarized test pulse to 0 mV to elicit peak *I*_Na_ (Figure 1F). Normalized I_Na_/I_Na_max values reflected the fraction of Na_v_ channels that were inactivated during the prepulse. The inactivation curves of Na_v_ recorded in PV^+^ neurons were in a significant depolarized direction compared with pyramidal neurons (bUsing a two-way ANOVA, *P* < 0.001 for interaction between groups × time, *F*_(11,209)_ = 17.73; *P* < 0.001 for time, *F*_(2.201, 41.83)_ = 1,147; *P* < 0.001 for PV^+^ vs. pyramidal neurons, *F*_(1,19)_ = 43.46; *n* = 5–17, Figure 1G). The V_1/2activation_ values for Na_v_ are the voltages at which the currents of inactivation are half-maximal. Therefore, the voltage-dependent inactivation of PV^+^ neurons was significantly depolarized compared with pyramidal neurons (−38.95 ± 2.74 vs. −47.72 ± 3.82 mV, *P* < 0.001, by unpaired *t*-test, *n* = 8–13, Figure 1H).

As neuronal firing frequency partly depends on how fast Na_v_ can cycle through their various states, including resting, activation, and/or inactivation, the recovery time from inactivation was recorded between PV^+^ neurons and pyramidal neurons. Consistent with voltage-dependent inactivation, recovery was also recorded by a double-pulse protocol. The peak *I*_Na_ was elicited in response to two 5-ms pulses at 0 mV, while the intervals between the two pulses ranged from 1 to 200 ms (Figure 1I). Time-dependent recovery curves of Na_v_ were significantly slower in pyramidal neurons compared with PV^+^ neurons at the physiological holding potential of −70 mV [by two-way ANOVA, *P* < 0.001 for interaction between groups × time, *F*_(11,264)_ = 49.77; *P* < 0.001 for time, *F*_(1.550, 37.20)_ = 1,158; *P* < 0.001 for PV^+^ vs. pyramidal neurons, *F*_(1,24)_ = 76.50; *n* = 12–15, Figure 1J]. The recovery time (Tau) of Na_v_ was accordingly faster in PV^+^ neurons than those in pyramidal neurons (2.96 ± 0.80 ms in PV^+^ neurons vs. 7.42 ± 2.01 ms in pyramidal neurons, *P* < 0.001 by unpaired *t*-test, *n* = 11–15, Figure 1K) from a holding potential of −70 mV. The above results indicate that the properties of Na_v_ in PV^+^ neurons lead to slower inactivation and faster recovery than those in pyramidal neurons.

To exactly describe the magnitude and the recovery time of the Na_v_ currents in pyramidal and PV^+^ neurons before and after compensation, we showed the Na_v_ currents before and after electronic compensation, which included the series resistance and capacitance transients. Then, with the perfusion of 200 nM TTX in the external solution, we found that TTX diminished the peak *I*_*Na*_ from −4,577.0 ± 1,668.0 pA to −36.52 ± 25.56 pA in pyramidal neurons (*P* < 0.001, *n* = 9, Supplementary Figure 1A) and from−5,341.0 ± 1,585.0 pA to −16.84 ± 5.69 pA in PV+ neurons (*P* < 0.001, *n* = 7, Supplementary Figure 1B). From these results, we identified that the recorded Na_v_ currents were all TTX-sensitive currents rather than capacitance currents.

### Isoflurane enhances voltage-dependent inactivation and delays the recovery time of Na_v_ on PV^+^ neurons in the PFC

**Figure 2:**
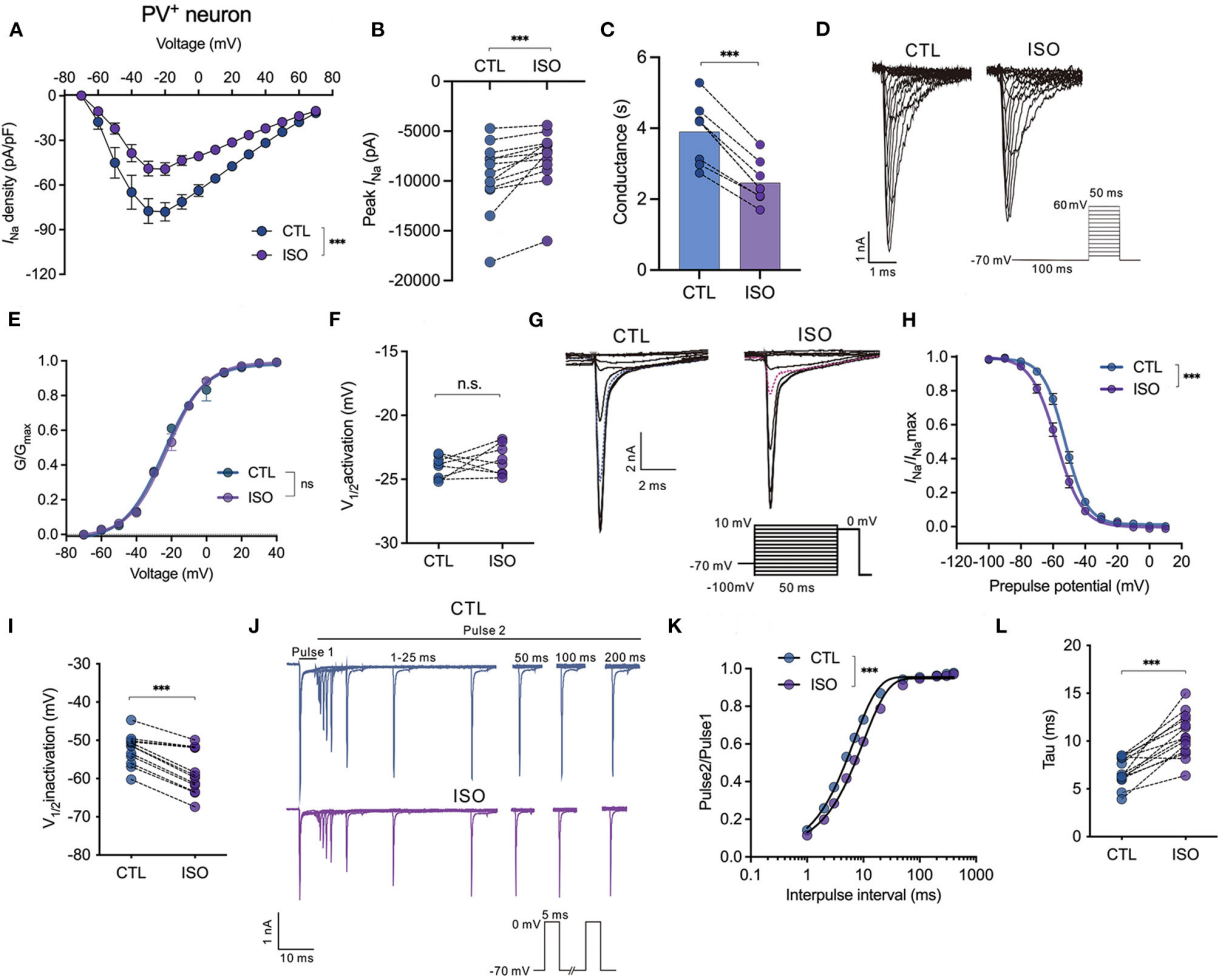
Effects of isoflurane on the gating properties of Na_v_ in PV^+^ neurons. **(A)** Isoflurane (~1.5 MAC) significantly inhibited the current-voltage (I-V) relationship (two-way ANOVA, *P* < 0.001 for interaction between groups × time, *F*_(14,196)_ = 4.630; *P* < 0.001 for time, *F*_(1.712, 23.96)_ = 70.35; *P* = 0.002 for control vs. isoflurane, *F*_(1,14)_ = 14.64; *n* = 10 cells from five mice). **(B)** Isoflurane significantly inhibited the peak amplitude of the current of Na_v_ at −70 mV holding potential. **(C)** Isoflurane significantly reduced the conductance of Na_v_ at a holding potential of −70 mV. **(D, E)** A representative trace for the activation of Na_v_ in response to −70 to +60 mV **(D)** and activation curves for Na_v_ **(E)** before and after exposure to ~1.5 MAC isoflurane (*n* = 8 cells from 4 to 5 mice). **(F)** Isoflurane did not significantly affect the voltage of half-maximum activation (V_1/2activation_) (*n* = 8 cells from 4 to 5 mice). **(G, H)** A representative trace for the voltage-dependent inactivation of Na_v_ in response to −100 to +10 mV by a double pulse **(G)** and inactivation curves for Na_v_ **(H)** under exposure to ~1.5 MAC isoflurane. **(I)** Isoflurane shifted the voltage dependence of the inactivation of Na_v_ in a hyperpolarized direction (−52.43 ± 4.05 vs. −58.35 ± 5.71 mV, *P* < 0.001, by unpaired *t*-test, *n* = 12 cells from 6 to 7 mice). **(J)** A representative trace for the recovery of Na_v_ before and after exposure to ~1.5 MAC isoflurane. **(K, L)** Isoflurane significantly increased the full channel recovery time of Na_v_ **(K)** and recovery time constant (tau) from inactivation **(L)** (6.80 ± 1.47 ms vs. 10.62 ± 2.26 ms, *P* < 0.001, using the paired *t*-test, *n* = 14 cells from seven mice) at a holding potential of −70 mV. Data are presented as means ± SEM. ***P < 0.001; n.s., not significant.

The effects of isoflurane at a clinically relevant concentration of ~1.5 MAC on Na_v_ activation, inactivation, recovery, and peak currents (*I*_Na_) were recorded and compared between pyramidal neurons and PV^+^ neurons in PFC. First, isoflurane at ~1.5 MAC significantly inhibited the current-voltage (I-V) relationship (by two-way ANOVA, *P* < 0.001 for interaction between groups × time, *F*_(14,196)_ = 4.630; *P* < 0.001 for time, *F*_(1.712, 23.96)_ = 70.35; *P* = 0.002 for control vs. isoflurane, *F*_(1,14)_ = 14.64; *n* = 10, Figure 2A). Further, isoflurane at ~1.5 MAC significantly decreased the current amplitude of Na_v_ (from −9,561.0 ± 3,454.0 pA to −7,734.0 ± 2,908.0 pA, *P* = 0.0002 by Wilcoxon test, *n* = 13, Figure 2B) and suppressed the conductance of Na_v_ (from 3.90 ± 0.87 ms to 2.46 ± 0.59 ms, *P* < 0.001 by paired *t*-test, *n* = 6, Figure 2C) at −70 mV holding potential. While isoflurane did not affect the voltage-dependent activations [by two-way ANOVA, *P* = 0.608 for interaction between groups × time, *F*_(11,198)_ = 0.832; *P* < 0.001 for time, *F*_(4.424, 79.62)_ = 12,344; *P* = 0.007 for control vs. isoflurane, *F*_(1,18)_ = 0.737, *n* = 10, Figures 2D, E] nor the voltages of half-maximal activation (V_1/2activation_) of Na_v_ in PV^+^ neurons (V_1/2activation_ control = −24.06 ± 0.87 mV; V_1/2activation_ isoflurane = −23.48 ± 1.18 mV, *P* = 0.284 by paired *t*-test, *n* = 8, Figure 2F). Next, isoflurane significantly shifted the voltage-dependence of steady-state inactivation of Na_v_ in a hyperpolarizing direction (Figures 2G, H) and significantly hyperpolarized the V_1/2inactivation_ from −52.43 ± 4.05 to −58.35 ± 5.71 mV (*P* < 0.001, by paired *t*-test, *n* = 12, Figure 2I). Moreover, isoflurane also significantly increased the full channel recovery time of Na_v_ in PV^+^ neurons [using a two-way ANOVA, *P* < 0.001 for interaction between groups × time, *F*_(11,308)_ = 4.630; *P* < 0.001 for time, *F*_(1.587, 44.44)_ = 3,083; *P* = 0.002 for control vs. isoflurane, *F*_(1,28)_ = 11.82; *n* = 15, Figures 2J, K] and slowed the recovery time (Tau) of Na_v_ from 6.80 ± 1.47 ms to 10.62 ± 2.26 ms at −70 mV holding potential (*P* < 0.001, by paired *t*-test, *n* = 14, Figure 2L) in PV^+^ neurons. These results indicate that for PV^+^ neurons, isoflurane at clinical concentration led to a decrease in the peak current of *I*_Na_ and a delay in the recovery from the inactivation state by increasing the fraction of inactivated Na_v_ at physiological resting membrane potentials.

### Isoflurane enhances voltage-dependent inactivation and delays the recovery time of Na_*v*_ on pyramidal neurons in the PFC

**Figure 3:**
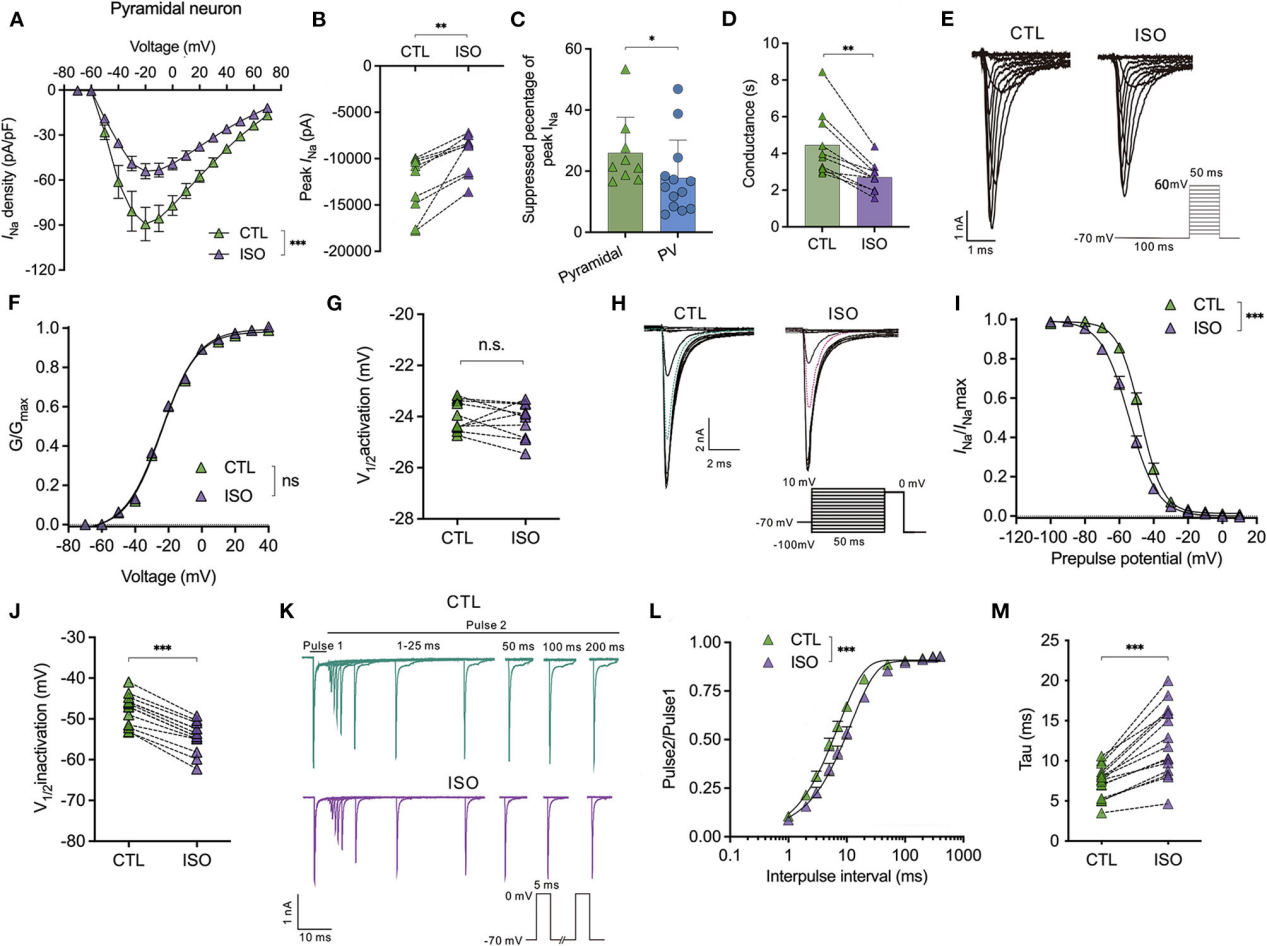
Effects of isoflurane on the gating properties of Na_v_ in pyramidal neurons. **(A)** Isoflurane (~1.5 MAC) significantly inhibited the current-voltage (I-V) relationship of Na_v_ in pyramidal neurons (using a two-way ANOVA, *P* < 0.001 for interaction between groups × time, *F*_(14,252)_ = 4.704; *P* < 0.001 for time, *F*_(1.364, 24.55)_ = 76.91; *P* = 0.002 for control vs. isoflurane, *F*_(1,18)_ = 13.32; *n* = 10 cells from six mice). **(B)** Isoflurane significantly inhibited the peak amplitude of the current of Na_v_ at −70 mV holding potential. **(C)** Suppressed percentage of the current amplitude of Na_v_ was significantly larger in pyramidal neurons than that in PV^+^ neurons (*P* = 0.032 by Mann-Whitney test, *n* = 9–13 from 5 to 6 mice). **(D)** Isoflurane significantly depressed the conductance of Na_v_ at a holding potential of −70 mV. **(E, F)** A representative trace for the activation of Na_v_ in response to −70 to +60 mV **(E)** and activation curves for Na_v_ **(F)** before and after exposure to ~1.5 MAC isoflurane (*n* = 10 cells from 5 to 6 mice). **(G)** Isoflurane did not significantly affect the voltage of half-maximum activation (V_1/2activation_) (*n* = 10 cells from 5 to 6 mice). **(H)** A representative trace for the voltage-dependent inactivation of Na_v_ in response to −100 to +10 mV by double pulse under exposure to ~1.5 MAC isoflurane. **(I)** Isoflurane shifted the voltage dependence of the inactivation of Na_v_ in a hyperpolarized direction (*P* < 0.001, by paired *t*-test, *n* = 13 from 6 to 7 mice). **(J)** Isoflurane significantly inhibits the voltage of half-maximum inactivation (V_in1/2activation_) of pyramidal neurons (*P* < 0.001, using the paired *t*-test, *n* = 13 from 6 to 7 mice). **(K)** A representative trace for the recovery of Na_v_ before and after exposure to ~1.5 MAC isoflurane. **(L, M)** Isoflurane significantly increased the full channel recovery time of Na_v_ **(I)** [two-way ANOVA, *P* < 0.001 for interaction between groups × time, *F*_(11,308)_ = 10.84; *P* < 0.001 for time, *F*_(1.425, 39.89)_ = 1,478; *P* = 0.022 for control vs. isoflurane, *F*_(1,28)_ = 5.913; *n* = 15] and recovery time constant (tau) from inactivation **(J)** (*P* < 0.001, using the paired *t*-test, *n* = 25). Data are presented as means ± SEM. *P < 0.05, **P < 0.01, ***P < 0.001; n.s., not significant.

For the Na_v_ in pyramidal neurons, isoflurane significantly inhibited the current-voltage curve [using a two-way ANOVA, *P* < 0.001 for interaction between groups × time, *F*_(14,252)_ = 4.704; *P* < 0.001 for time, *F*_(1.364, 24.55)_ = 76.91; *P* = 0.002 for control vs. isoflurane, *F*_(1,18)_ = 13.32; *n* = 10, Figure 3A] and significantly reduced the current amplitude of Na_v_ (from −12,985.0 ± 3,191.0 pA to −9,454.0 ± 2,223.0 pA, *P* = 0.002 using the paired *t*-test, *n* = 9, Figure 3B] at a holding potential of −70 mV. The suppressed percentage of the current amplitude of Na_v_ was calculated as (control peak *I*_*Na*_– isoflurane peak *I*_*Na*_)/control peak *I*_*Na*_. As a result, the suppressed percentage of the current amplitude of Na_v_ was significantly larger in pyramidal neurons compared to that in PV^+^ neurons (25.99 ± 11.63% vs. 17.78 ± 12.49%, *P* = 0.032 using the Mann–Whitney test, *n* = 9–13, Figure 3C). Moreover, ~1.5 MAC isoflurane also significantly suppressed the conductance of Na_v_ (from 4.46 ± 1.76 ms to 2.71 ± 0.79 ms, *P* = 0.002 using the Wilcoxon test, *n* = 9, Figure 3D) at the holding potential of −70 mV in pyramidal neurons.

However, it rarely affects the voltage-dependent activation [using a two-way ANOVA, *P* = 0.608 for interaction between groups × time, *F*_(11,198)_ = 0.832; *P* < 0.001 for time, *F*_(4.424, 79.62)_ = 12,344; *P* = 0.007 for control vs. isoflurane, *F*_(1,18)_ = 0.737; *n* = 10, Figures 3E, F] and does not affect the voltage-dependence of half-maximal activation (V_1/2activation_) of Na_v_ in pyramidal neurons (V__1/_2activation_ control= −23.98 ± 0.60 mV; V_1/2activation_ isoflurane= −24.16 ± 0.70 mV, *P* = 0.536 using the paired *t*-test, Figure 3G). Nevertheless, isoflurane significantly shifted the voltage dependence of steady-state inactivation (Figures 3H, I) in a hyperpolarizing direction from −47.72 ± 3.82 to −54.43 ± 3.76 mV (*P* < 0.001, using the paired *t*-test, *n* = 13, Figure 3J). In addition, isoflurane increased the full channel recovery time [using a two-way ANOVA, *P* < 0.001 for interaction between groups × time, *F*_(11,308)_ = 10.84; *P* < 0.001 for time, *F*_(1.425, 39.89)_ = 1,478; *P* = 0.022 for control vs. isoflurane, *F*_(1,28)_ = 5.913; *n* = 15, Figures 3K, L] and slowed the recovery time (Tau) from 7.42 ± 2.01 ms to 12.40 ± 4.34 ms at a physiological holding potential of −70 mV (*P* < 0.001, by paired *t*-test, *n* = 25, Figure 3M) in pyramidal neurons. These results indicate that, for pyramidal neurons, isoflurane at clinical concentration led to a decrease in the peak *I*_Na_ and a delay in the recovery time from the inactivation state by increasing the fraction of inactivated Na_v_ at physiological resting membrane potentials.

### Isoflurane inhibits peak sodium currents in pyramidal neurons more potently than those in PV^+^ neurons at a physiological holding potential

**Figure 4:**
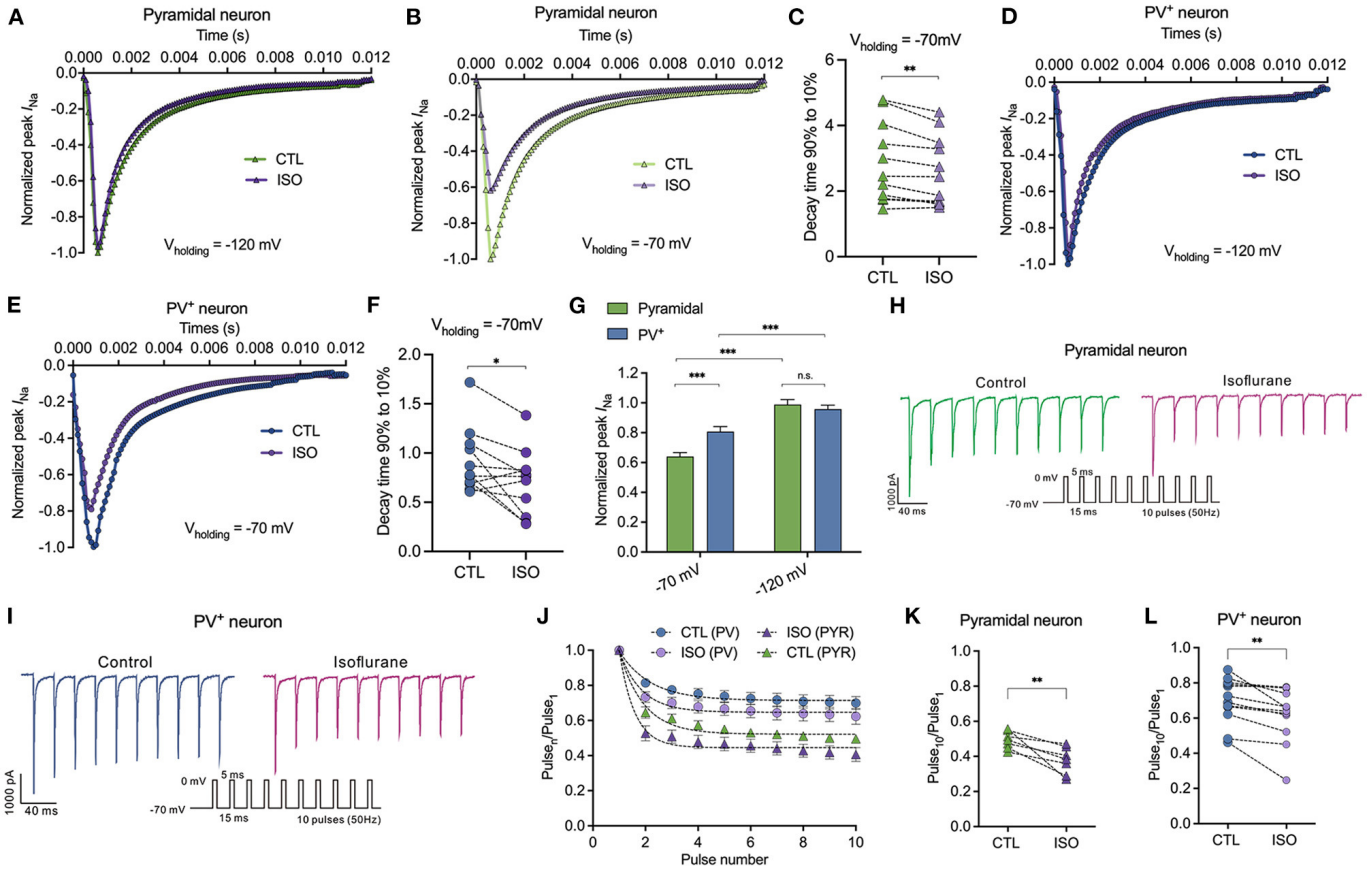
Effects of isoflurane on peak Na current of Na_v_ in pyramidal neurons and PV^+^ neurons at a physiological and hyperpolarizing holding potential. **(A, B)** ~1.5 MAC isoflurane did not inhibit peak INa of pyramidal neurons at V_h_ = −120 mV (*n* = 6 cells from 4 to 5 mice) **(A)** but did inhibit a significant reduction of those neurons at V_h_ = −70 mV (*n* = 11 cells from 5 to 6 mice) **(B)**. **(C)** ~1.5 MAC isoflurane significant suppressed 90–10% decay time of Na_v_ in pyramidal neurons (*n* = 11 cells from 6 to 7 mice). **(D, E)** ~1.5 MAC isoflurane did not inhibit peak *I*_Na_ of PV^+^ neurons at V_h_ = −120 mV (*n* = 5 cells from 4 to 5 mice) **(D)**, whereas a significant reduction of those neurons at V_h_ = −70 mV (*n* = 5 cells from 4 to 5 mice) **(E)**. **(F)** ~1.5 MAC isoflurane significantly suppressed 90–10% decay time of Na_v_ in PV^+^ neurons (*n* = 11 cells from 6 to 7 mice). **(G)** ~1.5 MAC isoflurane suppressed peak *I*_Na_ more potently in pyramidal neurons than those in PV^+^ neurons at a holding potential of −70 mV (*n* = 5–1 cells from 4 to 6 mice). **(H, I)** A representative trace for the repeated pulses of depolarizations in pyramidal neurons **(H)** and/or in PV+ neurons **(I)**. **(J)** Peak *I*_*Na*_ elicited by a 50 Hz train of 5-ms pulses before and after exposure to isoflurane between pyramidal neurons and PV^+^ neurons with a holding potential at −70 mV. **(K, L)** Normalized *I*_*Na*_ at Pulse_10_ in pyramidal neurons **(K)** or PV^+^ neurons **(L)**. Data are presented as means ± SEM. *P < 0.05, **P < 0.01, ***P < 0.001; n.s., not significant.

All the above results indicate that ~1.5 MAC isoflurane significantly prolonged the steady-state inactivation of Na_v_ at physiological holding potentials. Therefore, in this study, we examined the distinct sensitivities of ~1.5 MAC isoflurane on peak *I*_Na_ inhibition between PV^+^ and pyramidal neurons. First, the percentage of inhibition on peak *I*_Na_ by isoflurane was recorded from a holding potential of −120 and/or −70 mV, respectively. With the holding potential at −120 mV, isoflurane did not inhibit peak *I*_Na_ in PV^+^ or pyramidal neurons (Figures 4A, D, G). At the physiological holding potential of −70 mV, isoflurane significantly inhibited the peak *I*_Na_ in pyramidal neurons by 35.95 ± 13.32% and by 19.24 ± 16.04% in PV^+^ neurons (*P* < 0.001 for both PV^+^ and pyramidal neurons, using the Mann–Whitney test, Figures 4B, E, G). When comparing the inhibitions of peak *I*_Na_ by isoflurane between PV^+^ and pyramidal neurons at a holding potential of −70 mV, isoflurane suppressed peak *I*_Na_ more potently in pyramidal neurons than those in PV^+^ neurons (*P* < 0.001, using the Mann–Whitney test, Figure 4G). Since ~1.5 MAC isoflurane significantly suppressed the peak *I*_Na_ at a holding potential of −70 mV both in pyramidal and PV^+^ neurons, we analyzed the kinetics of Na_v_ at the voltage step of −20 mV, which corresponded to the recording of peak *I*_Na_. Isoflurane at ~1.5 MAC significantly inhibited the 90–10% decay time (from 2.86 ± 1.22 ms to 2.62 ± 1.06 ms in pyramidal neurons, *P* = 0.004 using the paired *t*-test, Figure 4C; from 0.918 ± 0.33 ms to 0.71 ± 0.33 ms in PV^+^ neurons, *P* = 0.024 using the paired *t*-test, Figure 4F), which ultimately slowed the recovery time of Na_v_. As a result, isoflurane accelerated the decay phases, which was consistent with the effects of isoflurane on transfected Nav (Zhou et al., 2019).

The slow recovery from inactivation after membrane depolarization would lead to progressive inhibition of *I*_*Na*_ during trains of action potentials. With repeated 5-ms depolarizing pulses to 0 mV from a holding potential of −70 mV at 50 Hz (Figures 4H, I), the peak *I*_*Na*_ of each pulse normalized to that of the first pulse (Pulse_n_/Pulse_1_) (Figure 4J) to remove the effect of the resting block by isoflurane in PV^+^ and pyramidal neurons. Therefore, the reduced *I*_*Na*_ at the 10th pulse reflected activity-dependent inhibition as a result of repeated membrane depolarization. Isoflurane reduced the pulse_10_/pulse_1_ ratio from 0.49 ± 0.05 to 0.38 ± 0.08 (*P* = 0.009 by paired *t*-test, *n* = 7, Figure 4K) in pyramidal neurons and from 0.70 ± 0.13 to 0.62 ± 0.16 (*P* = 0.004 by paired *t*-test, *n* = 12, Figure 4L) in PV^+^ neurons.

### Isoflurane differentially inhibits the amplitudes of action potentials between PV^+^ and pyramidal neurons in the PFC

**Figure 5:**
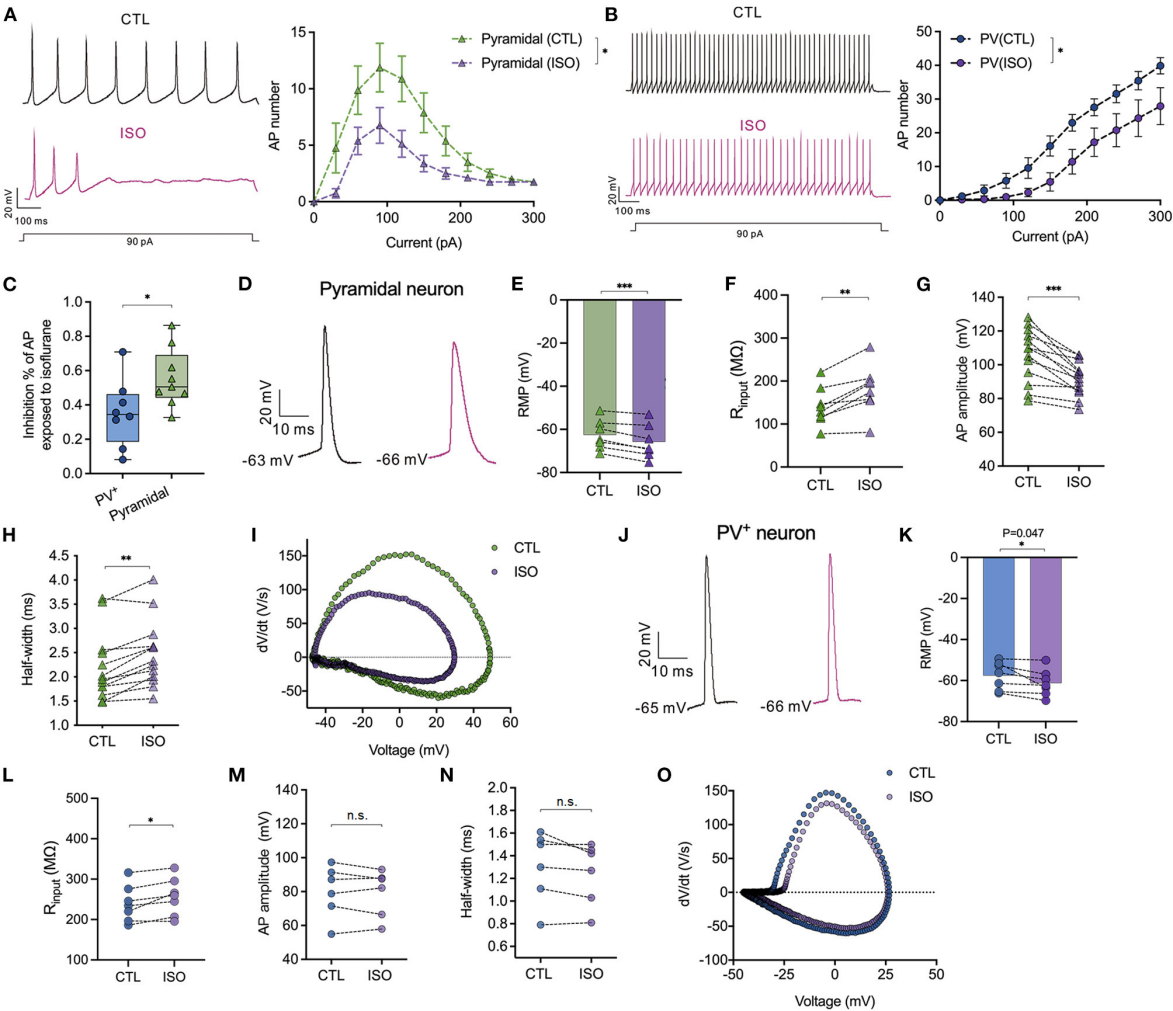
Effects of isoflurane on action potentials of PV^+^ and pyramidal neurons. **(A)** The left panel showed the representative trace of action potentials in response to a depolarizing current (1,000 ms, 90 pA) injection for pyramidal neurons; the right panel showed the inhibitory effects of ~1.5 MAC isoflurane on the action potential frequency when injecting depolarizing currents from 0 to 300 pA for pyramidal neurons (*n* = 9 cells from 5 to 6 mice). **(B)** The left panel showed the representative trace of action potentials in response to a depolarizing current (1,000 ms, 90 pA) injection for PV^+^ neurons; the right panel showed the inhibitory effects of ~1.5 MAC isoflurane on the action potential frequency when injecting depolarizing currents from 0 to 300 pA for PV^+^ neurons (*n* = 8 cells from 5 to 6 mice). **(C)** Isoflurane inhibited the AP frequency more potently in pyramidal neurons than PV^+^ neurons (55.48 ± 16.96% vs. 35.33 ± 19.49%, *P* = 0.036 by Mann-Whitney test, *n* = 8–9 from 5 to 6 mice). The suppressed percentage of area under the curve (AUC) for the AP frequency-current relationship was calculated as (control AUC – isoflurane AUC)/control AUC. **(D)** Representative traces of the first evoked AP of pyramidal neurons. **(E, F)** ~1.5 MAC isoflurane significantly decreased the RMP (*P* = 0.001 by paired *t*-test, *n* = 7 cells from 4 to 5 mice) **(E)** and increased R_in_ (*P* = 0.003 by paired *t*-test, *n* = 8 cells from five mice) **(F)** of pyramidal neurons. **(G, H)** The analysis of AP amplitude **(G)** and AP half-width **(H)** (*n* = 13 cells from 6 to 7 mice) of pyramidal neurons. **(I)** Representative traces for dv/dt of the first evoked AP of pyramidal neurons. **(J)** Representative traces of the first evoked AP of PV^+^ neurons. **(K, L)** ~1.5 MAC isoflurane significantly decreased the RMP (*P* = 0.047 using the paired *t*-test, *n* = 7 cells from 4 to 5 mice) **(K)** and increased R_in_ (*P* = 0.019 using the paired *t*-test, *n* = 7 cells from 4 to 5 mice) **(L)** of PV^+^ neurons. **(M, N)** The analysis of AP amplitude **(M)** and AP half-width **(N)** of PV^+^ neurons (*n* = 6 cells from 5 to 6 mice). **(O)** Representative traces for dv/dt of the first evoked AP of PV^+^ neurons. Data are presented as means ± SEM. *P < 0.05, **P < 0.01, ***P < 0.001; n.s., not significant.

The effects of isoflurane (~1.5 MAC) on the neuronal excitability of PV^+^ and pyramidal neurons were evaluated in PFC. When subjected to ~1.5 MAC isoflurane, the action potential frequency was significantly suppressed while injecting depolarizing currents from 0 to 300 pA on both pyramidal neurons [by two-way ANOVA, *P* < 0.001 for interaction between groups × time, *F*_(10,200)_ = 3.710; *P* < 0.001 for time, *F*_(2.482, 49.64)_ = 22.97; *P* < 0.001 for control vs. isoflurane, *F*_(1,20)_ = 16.50, *n* = 15, Figure 5A] and/or PV^+^ neurons [by two-way ANOVA, *P* = 0.046 for interaction between groups × time, *F*_(10,160)_ = 1.923; *P* < 0.001 for time, *F*_(2.240, 35.83)_ = 81.88; *P* = 0.01 for control vs. isoflurane, *F*_(1,16)_ = 8.506, *n* = 9, Figure 5B]. The suppressed percentage of area under the curve (AUC) of the AP frequency-current was calculated as (control AUC – isoflurane AUC)/control AUC. As a result, the suppressed percentage of the AUC of the AP frequency was significantly larger in pyramidal neurons compared to that in PV^+^ neurons (55.48 ± 16.96% vs. 35.33 ± 19.49%, *P* = 0.036 using the Mann–Whitney test, Figure 5C). When subjected to ~1.5 MAC isoflurane, the RMPs of pyramidal (Figure 5D) and PV^+^ neurons (Figure 5J) were significantly decreased (−62.55 ± 6.93 mV to −65.80 ± 7.81 mV in pyramidal neurons, *P* = 0.001 using the paired *t*-test, *n* = 7, Figure 5E; −57.56 ± 6.79 mV to −61.21 ± 6.47 mV in PV^+^ neurons, *P* = 0.047 using the paired *t*-test, *n* = 7, Figure 5K) and were accompanied by an increase in R_in_ of pyramidal and PV^+^ neurons (141.20 ± 44.25 MΩ vs. 180.70 ± 56.22 MΩ in pyramidal neurons, *P* = 0.003 using the paired *t*-test, *n* = 8, Figure 5F; 239.20 ± 45.32 MΩ vs. 256.60 ± 46.63 MΩ in PV^+^ neurons, *P* = 0.019 using the paired *t*-test, *n* = 7, Figure 5L).

The properties of APs, including amplitude and half-width, were analyzed. Isoflurane at ~1.5 MAC significantly reduced the amplitudes of the first AP in pyramidal neurons (105.9 ± 15.89 mV vs. 91.55 ± 10.16 mV, *P* < 0.001, using the paired *t*-test, *n* = 13, Figure 5G), but it did not affect the AP amplitudes in PV^+^ neurons (80.19 ± 15.36 mV vs. 79.17 ± 13.86 mV, *P* = 0.544, using the paired *t*-test, *n* = 6, Figure 5M). Similarly, isoflurane (~1.5 MAC) increased the half-width of APs in pyramidal neurons from 2.19 ± 0.70 ms vs. 2.48 ± 0.69 ms, *P* = 0.001, using the Wilcoxon test, *n* = 13, Figures 5H, I) but did not affect the half-width of APs in PV^+^ neurons (1.31 ± 0.31 ms vs. 1.25 ± 0.27 ms, *P* = 0.105 using the paired *t*-test, *n* = 6, Figures 5N, O).

### Effects of isoflurane on simulated APs and synaptic transmission in pyramidal and PV^+^ neurons

**Figure 6:**
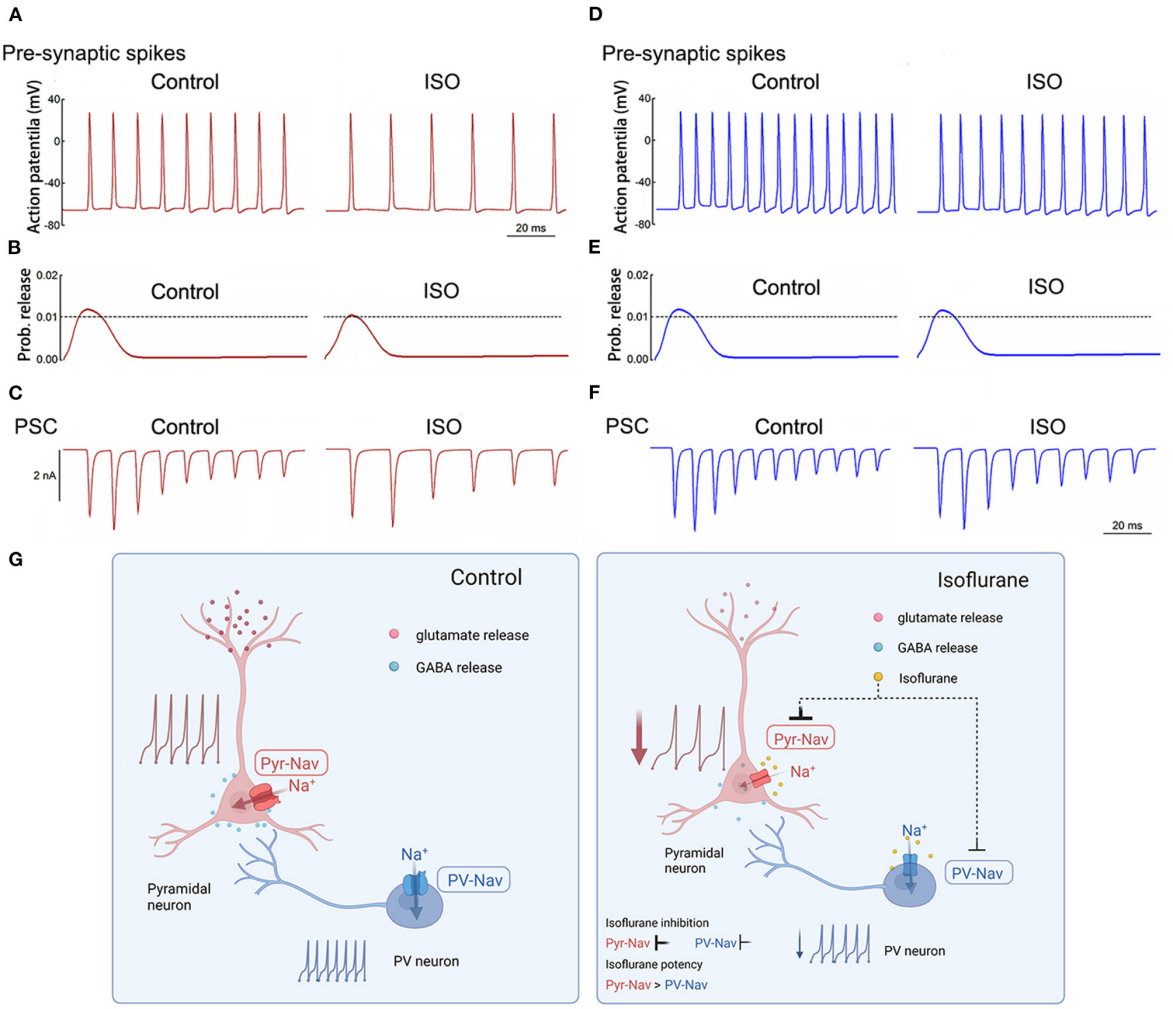
Simulated effects of isoflurane on action potentials and synaptic transmission in pyramidal and PV^+^ neurons. **(A)** Effects of isoflurane on simulated AP frequency evoked by a long stimulus of 0.5 nA for 250 milliseconds for pyramidal neurons. **(B)** The relationship between AP amplitude and the probability of transmitter release was modified from an established nerve terminal model for pyramidal neurons. **(C)** Simulated effects of isoflurane on synaptic transmission in pyramidal neurons for pyramidal neurons. **(D)** Effects of isoflurane on simulated AP frequency evoked by a long stimulus of 0.5 nA for 250 ms for pyramidal neurons. **(E)** The relationship between AP amplitude and the probability of transmitter release was modified from an established nerve terminal model for pyramidal neurons. **(F)** Simulated effects of isoflurane on synaptic transmission in pyramidal neurons for pyramidal neurons. **(G)** Summary of presynaptic mechanisms contributing to differential suppression of glutamate release and GABA release. Left: the excitatory-inhibitory circuits in the cortex under control condition; right: the Na_v_ of pyramidal neurons is more sensitive to isoflurane than that of PV^+^ neurons, which induces different inhibition between glutamate release and GABA release, resulting in the net depression of excitatory-inhibitory circuits in the cortex.

The NEURON algorithm was used to simulate the effects of isoflurane on presynaptic AP and synaptic currents that were mediated by its modulations on AP firing frequencies and properties. Presynaptic APs were evoked by a long depolarizing current (250 ms current injection at 0.5 nA, Figures 6A, D). The baseline APs frequency evoked in pyramidal neurons was lower than that in PV^+^ neurons by adjusting the parameters of the model to lead to an AP frequency similar to the recorded AP frequency in patch-clamping experiments. The probability of neurotransmitter release was based on the amplitudes of presynaptic APs (Graham and Redman, 1994). Post-synaptic currents were determined by both presynaptic AP frequency and As amplitudes and were assumed to be both excitatory transmissions. The effects of isoflurane in this simulation were based on our previous recordings in brain slices. Isoflurane demonstrated an inhibition of AP firing frequency of 35% in pyramidal neurons and 20% in PV^+^ neurons. Additionally, it resulted in a reduction of 14% in AP amplitude for pyramidal neurons, while no significant effect was observed for PV^+^ neurons.

In the above-described simulations, the probability of neurotransmitter release and/or post-synaptic currents was preferentially suppressed by isoflurane in pyramidal neurons (Figures 6B, C) than in PV^+^ neurons (Figures 6E, F). Therefore, isoflurane differentially inhibits Na_v_ currents between pyramidal and PV^+^ neurons in the cortex, which may contribute to the preferential suppression of glutamate release over GABA release, resulting in the net depression of excitatory-inhibitory circuits in the PFC (Figure 6G).

## Discussion

The present study, which combined electrophysiological recording and simulation *in silico*, reveals that isoflurane inhibits Na_v_ currents in pyramidal neurons more potently than PV^+^ neurons in the PFC. First, we found that the voltage-dependent gating properties of the Na_v_ channel vary between PV^+^ neurons and pyramidal neurons, as evidenced by the similarity of the voltage-dependent activations of Na_v_ between the two cellular subtypes, while their steady-state inactivation and recovery time are significantly different. Second, the effects of isoflurane at clinically relevant concentrations facilitated the voltage-dependent inactivation in a hyperpolarized direction and delayed the recovery time of Na_v_, and these effects were more potent in pyramidal neurons than those in PV^+^ neurons. Third, the differential modulations of isoflurane on neuronal excitability between PV^+^ and pyramidal neurons may result from the varied sensitivity of isoflurane to the Na_v_ subtypes that are differentially expressed in these two neuronal subtypes.

Neuronal action potentials and synaptic transmission vary between brain regions and cellular subtypes for both physiologic and pharmacological conditions, which may be derived from the differential expression of Na_v_ subtypes in extent. Among the nine determined subtypes of the Na_v_ channel, Na_v_1.1, Na_v_1.2, and Na_v_1.6 are the most abundantly expressed subtypes in the central nervous system. There are Na_v_ subtypes with distinct cellular and subcellular distributions. For example, hippocampal and cortical PV^+^ neurons are enriched with Na_v_1.1 mostly in their axons and the initial axonal segment, which controls the axonal excitability and determines AP initiation and propagation (Hu and Jonas, 2014; Li et al., 2014). Otherwise, hippocampal and cortical glutamatergic neurons, typically pyramidal neurons, are more abundant with Na_v_1.6 in axons and the initial axonal segment (Speigel and Hemmings, 2021). The neuronal Na_v_ subtypes are distinct in their voltage-dependent gating properties, which are crucial to mediating neuronal excitability, including the initiation and propagation of action potentials (APs). In the present study, the voltage-dependent activations of Na_v_ were similar between pyramidal and PV^+^ neurons, while their voltage-dependent inactivation and recovery time was different between the two neuronal subtypes. Previously, the voltage-dependent inactivation of Na_v_1.1 was more depolarized than that of Na_v_1.6 and/or Na_v_1.2 in transfected cells (Zhou et al., 2019). In this study, the recorded voltage-dependent inactivation of Na_v_ was more depolarized in PV^+^ neurons compared to pyramidal neurons, which is consistent with the enriched expression of Na_v_1.1 in PV^+^ neurons compared with the enriched expression of Na_v_1.6 in pyramidal neurons (Speigel and Hemmings, 2021). In addition, since the V_1/2inactivaiton_ of Na_v_ was more depolarized in PV^+^ neurons than that in pyramidal neurons, there was a higher fraction of Na_v_ in the inactivated state under physiological potentials, which led to greater inhibition of Na_v_ currents in pyramidal neurons than that in PV^+^ neurons at a similar holding potential. Moreover, the differential expressions of Na_v_ subtypes may be neural substrates for the varied gating properties of Na_v_ and neurotransmitter release between PV^+^ neurons and pyramidal neurons.

Synaptic neurotransmission emerged as a main target for the pharmacological actions of volatile anesthetics, which mainly include the release of presynaptic neurotransmitters and the postsynaptic ligand-gated receptors (Hao et al., 2020). Interestingly, Baumgart et al. (2015) found that the inhibitory effect of isoflurane on synaptic vesicle exocytosis preferentially occurred in glutamatergic synapses compared with GABAergic synapses in an AP-dependent manner, which implied that at least one of the key molecular players controlling AP-driven neurotransmitter release was different in these two neuronal subtypes. A previous study has demonstrated that the inhibitory effects of isoflurane on glutamate release in glutamatergic synapses are more potent than those of GABA release in PV^+^ synapses in cultured hippocampal neurons, which partly relied on the differential expressions of Na_v_ subtypes in those neurons (Speigel and Hemmings, 2021). In this study, we focused on the anesthetic actions of isoflurane on Na_v_ between neuronal subtypes and found that isoflurane at ~1.5 MAC suppressed peak *I*_Na_ more potently in pyramidal neurons than those in PV^+^ neurons, which indicates that glutamatergic neurons are more sensitive to isoflurane than PV^+^ interneurons. This result is also consistent with previous studies showing that Na_v_1.2 and/or 1.6 are more sensitive to isoflurane than Na_v_1.1 at physiological membrane potentials (Zhou et al., 2019). In the present study, isoflurane at ~1.5 MAC did not affect the activation phase but accelerated the decay, which induced the enhancement of the voltage-dependent inactivation and delay of the recovery time of Na_v_ both in pyramidal and PV^+^ neurons. As previously reported, the effects of isoflurane on voltage-dependent activation of Na_v_ were similar between Na_v_1.1, Na_v_1.2, and/or Na_v_1.6, while their voltage-dependent inactivation was more depolarized in Na_v_1.1 compared with Na_v_1.2 and/or Na_v_1.6 (Zhou et al., 2011). In addition, isoflurane inhibited the peak *I*_Na_ with high voltage dependence, showing that none of the Na_v_ currents in PV^+^ or in pyramidal neurons were inhibited by isoflurane at a hyperpolarized holding potential (−120 mV). However, at the physiological holding potential (−70 mV), Na_v_ currents were both inhibited by isoflurane in those neuronal subtypes. This result is consistent with a low affinity of isoflurane for the resting state of Na_v_ (Purtell et al., 2015).

Neurotransmitter release is tightly coupled with the presynaptic Ca^2+^ influx through Ca_v_ that is activated by nerve terminal depolarization. Nevertheless, depolarization and Ca^2+^ entry are mainly regulated by the presynaptic ion channels, such as Na^+^, K^+^, and Ca^2+^ channels. AP-triggered Ca^2+^ entry is vigorously dependent on AP shape, particularly the falling phase (Clarke et al., 2016). Thus, small differences in presynaptic AP amplitude can produce large changes in the timing and magnitude of presynaptic Ca^2+^ entry because the kinetics of Ca^2+^ channels are non-linear (Clark et al., 1996). In the present study, isoflurane significantly decreased the AP amplitude of pyramidal neurons but rarely affected that of PV^+^ neurons, which may result in a less suppressed Ca^2+^ influx in PV^+^ neurons than that in pyramidal neurons. Based on these findings, the simulated AP frequency is higher in PV^+^ neurons than the AP frequency in pyramidal neurons, which is consistent with the fast spike property of PV^+^ interneurons (Brackenbury et al., 2010). In general, this study demonstrates that isoflurane may differentially inhibit Na_v_ currents between pyramidal and PV^+^ neurons in the PFC, which may contribute to the preferential suppression of glutamate release over GABA release within the PFC, resulting in a net depression of excitatory-inhibitory circuits in the PFC. Moreover, the depression of the PFC is the most important sign of unconsciousness induced by general anesthetics.

In this study, we recorded pyramidal neurons and PV^+^ neurons in layer 5 of the prefrontal cortex. It has been found that general anesthetics may downregulate the distal dendritic compartment in layer 5 pyramidal neurons (Meyer, 2015), and general anesthesia decouples the interaction between layer 5 pyramidal neuron dendrites and their soma in the mouse somatosensory cortex, which both contribute to the general anesthetic-induced unconsciousness (Suzuki and Larkum, 2020). All results indicate that the prefrontal cortex and layer 5 of the cortex are the key neural substrates relevant to unconsciousness induced by general anesthetics.

In the simulation part, the effects of isoflurane on the frequency of presynaptic neuronal spikes are based on the recorded suppression of isoflurane on AP frequency between pyramidal neurons and PV^+^ neurons. For the probability of neurotransmitter release, the simulation is based on the effects of isoflurane on AP height as previously reported (Speigel and Hemmings, 2021); therefore, at clinically relevant concentrations, isoflurane inhibits the probability of neurotransmitter release more potently in pyramidal neurons than PV^+^ neurons. At last, the simulation of post-synaptic currents is based on both the frequencies of presynaptic neuronal spikes and the probability of neurotransmitter release. As a result, isoflurane suppresses synaptic transmission more potently in pyramidal neurons than PV^+^ neurons, which produces a net inhibition in this excitatory-inhibitory microcircuit with the PFC.

This study also has some limitations. First, in the present study, we only observed the phenomenon that isoflurane differently inhibited the Na_v_ currents between pyramidal and PV^+^ neurons in cortex slices but did not identify the exact subtypes of Na_v_ between the neuronal subtypes. However, substantial studies have reported that PV^+^ neurons are enriched with Na_v_1.1 (Speigel and Hemmings, 2021), while glutamatergic neurons are highly expressed with Na_v_1.6, which may be the neuropharmacological basis for the selective sensitivity of isoflurane between the PV^+^ and glutamatergic neurons. Second, in this study, the Na_v_ currents and action potentials were recorded in neuronal soma by whole-cell patch clamping recordings, which reflect their characteristics in mainly cell soma but not in the presynaptic terminal region. Therefore, other methods, such as voltage-dependent imaging (Lamy and Chatton, 2011; Armbruster et al., 2022), may be useful for examining the effects of volatile anesthetics on presynaptic terminal firing and AP propagation between neuronal subtypes. Finally, for the whole-cell patch-clamping recording of fast currents in brain slices, due to the irregular shapes of the neurons, the space clamp limitation, as somatic voltage-clamp cannot effectively control voltages in cellular processes distant from the somatic recording site, may exist. Therefore, the different cellular arbors between pyramidal neurons and PV^+^ interneurons may partly account for some observations in this manuscript.

In conclusion, isoflurane differentially inhibits Na_v_ currents between pyramidal and parvalbumin neurons in the PFC, which may contribute to the preferential suppression of glutamate release over GABA release in the PFC. This effect of isoflurane leads to a net depression of excitatory-inhibitory circuits in the PFC.

## Data availability statement

The original contributions presented in the study are included in the article/Supplementary material, further inquiries can be directed to the corresponding authors.

## Ethics statement

The animal study was reviewed and approved by the Animal Ethics Committee of West China Hospital of Sichuan University.

## Author contributions

PL, TZ, and CZ: designed the research. JQ and YY: conducted the research. QL, WZ, and PL: analyzed data. YY, JQ, CZ, and JL: wrote the paper. All authors contributed to the article and approved the submitted version.

## Supplementary material

Supplementary Figure 1 A representative trace of Nav current in pyramidal and PV^+^ neurons before and after compensation and perfusion with TTX. **(A)** A representative trace of Na_v_ current in pyramidal neurons before and after compensation and perfusion with 200 nM TTX. **(B)** A representative trace of Na_v_ current in PV^+^ neurons before and after compensation and perfusion with 200 nM TTX.
