# Supplementary figures and images for: The volatile anesthetic isoflurane differentially inhibits voltage-gated sodium channel currents between pyramidal and parvalbumin neurons in the prefrontal cortex
Source: Front Neural Circuits. 2023 Jun 16;17:1185095. doi: 10.3389/fncir.2023.1185095 (PMC10311640; doi:10.3389/fncir.2023.1185095)

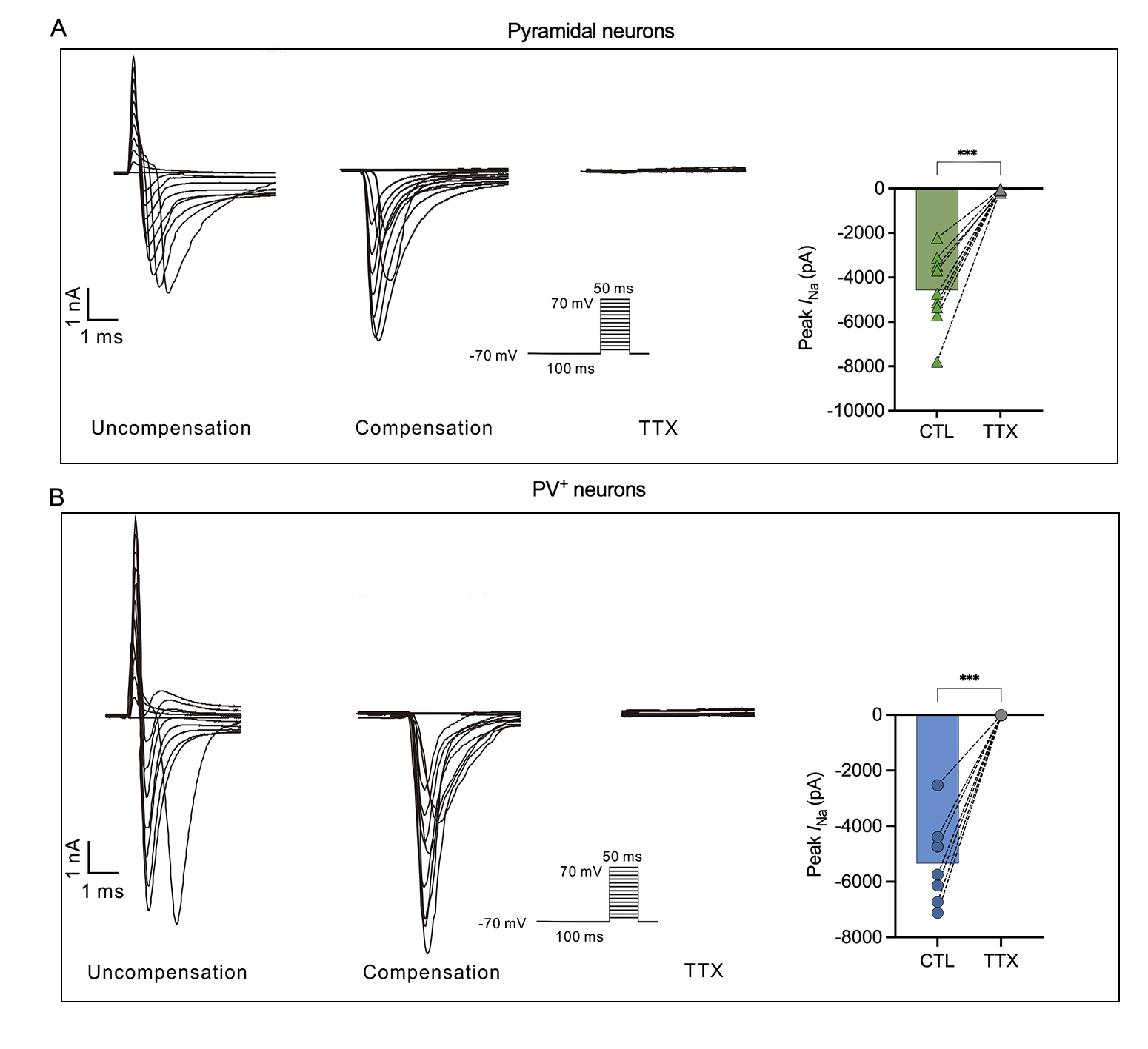

Supplement: Supplementary Figure 1 — A representative trace of Nav current in pyramidal and PV+ neurons before and after compensation and perfusion with TTX. (A) A representative trace of Nav current in pyramidal neurons before and after compensation and perfusion with 200 nM TTX. (B) A representative trace of Nav current in PV+ neurons before and after compensation and perfusion with 200 nM TTX. [file Image_1.tif]
